# GhERF.B4-15D: A Member of ERF Subfamily B4 Group Positively Regulates the Resistance against *Verticillium dahliae* in Upland Cotton

**DOI:** 10.3390/biom13091348

**Published:** 2023-09-05

**Authors:** Panpan Wang, Yanpeng Zhao, Na Wu, Muhammad Tehseen Azhar, Yuxia Hou, Haihong Shang

**Affiliations:** 1 Zhengzhou Research Base, National Key Laboratory of Cotton Bio-Breeding and Integrated Utilization, School of Agricultural Sciences, Zhengzhou University, Zhengzhou 450001, China; 2 National Key Laboratory of Cotton Bio-Breeding and Integrated Utilization, Institute of Cotton Research, Chinese Academy of Agricultural Sciences, Anyang 455000, China; 3 Department of Plant Breeding and Genetics, University of Agriculture, Faisalabad 38000, Pakistan; 4 College of Science, China Agricultural University, Beijing 100193, China

**Keywords:** *Verticillium dahliae*, upland cotton, ethylene response factor, GhERF.B4-15D, GhDREB1B

## Abstract

*Verticillium* wilt is a fungal disease in upland cotton and exerts a significant effect on growth and potential productivity. This disease is mainly caused by *V. dahliae* Kleb. Ethylene response factor (ERF) is one of the superfamilies of transcription factors that is involved in the development and environmental adaption of crops. A total of 30 ERF.B4 group members were detected in upland cotton and divided into 6 subgroups. Gene structures, conserved motifs, and domain analysis revealed that members in each subgroup are highly conserved. Further, the 30 GhERF.B4 group members were distributed on 18 chromosomes, and 36 gene synteny relationships were found among them. *GhERF.B4* genes were ubiquitously expressed in various tissues and developmental stages of cotton. Amongst them, *GhERF.B4-15D* was predominantly expressed in roots, and its expression was induced by *V. dahliae* infection. In addition, *GhERF.B4-15D* responded to methyl jasmonate (MeJA), methyl salicylate (MeSA), and ethylene (ET) phytohormones. It was also found that the *V. dahliae* resistance was enhanced due to overexpression of *GhERF.B4-15D* in *Arabidopsis thaliana*. On the contrary, interference of *GhERF.B4-15D* by virus-induced gene silencing (VIGS) technology decreased the *V. dahliae* resistance level in upland cotton. The subcellular localization experiment showed that GhERF.B4-15D was located in the nucleus. Yeast two-hybrid (Y2H) and luciferase complementation (LUC) approaches demonstrated that GhERF.B4-15D interacted with GhDREB1B. Additionally, the *V. dahliae* resistance was significantly decreased in GhDREB1B knockdowns. Our results showed that GhERF.B4-15D plays a role during *V. dahliae* infection in cotton.

## 1. Introduction

Upland cotton (*Gossypium hirsutum*) is one of the important natural fiber crops, in addition to its use as edible oil for humans and seedcake for livestock and fish [1]. However, *Verticillium* wilt (VW), which is mainly caused by the soil-borne fungi *V. dahliae*, is the main threat to the productivity of cotton [2]. Due to the lack of VW resistance in cotton germplasms, it was difficult to obtain resistance in existing accession through traditional breeding. However, various resistance mechanisms and functional genes were detected for cotton against *V. dahliae* infection [3]. Several defense mechanisms were involved in JA, SA, and ET signals, mitogen-activated protein kinase (MAPK) flow, or phosphorylation [3,4,5].

The AP2/ERF transcription factor family is one of the largest transcription factor families, containing a highly conserved AP2 domain that is composed of ~60 amino acids [6,7]. On the basis of the number of conserved domains and structural similarities of AP2, the AP2/ERF transcription factor family can be divided into five subfamilies, i.e., AP2, ERF, DREB, RAV, and Soloist [7]. Both DREB and ERF subfamilies contain only one AP2 domain, but their AP2 domain has two different amino acids [8]. Approximately 121 ERF subfamily members were detected in Arabidopsis, which were divided into 12 groups (A1 to A6, B1 to B6) [8]. However, 122 ERF subfamily members were identified in Arabidopsis, and AtERF109 was moved from the B3 group to the B4 group through phylogenetic analysis [9]. Therefore, there are eight members in the ERF subfamily B4 group, including AtERF108, AtERF109, AtERF110, AtERF111, AtERF112, AtERF113, AtERF114, and AtERF115. 

ERF plays an important role in plant growth, development, and environmental adaptation. Studies have shown that AP2/ERFs are widely involved in regulating stress responses mediated by ET, abscisic acid (ABA), gibberellin (GA), cytokinin CK, and brassinosteroids (BR) [10]. E3 ubiquitin ligase RGLG2 interacts with AtERF53, which negatively regulates plant responses to drought stress [11]. ERF5 and ERF6 activate the expression of stress-related transcription factor genes *STZ*, *WRKY33*, and *MYB51* [12]. The expression of *GhERF4L* and *GhERF54L* was upregulated under salt treatment, and silencing of *GhERF4L* and *GhERF54L* reduced the salt tolerance in cotton [13]. Meanwhile, studies revealed that ERF transcription factors are involved in innate immunity. ERF regulates *Pathogenesis-related* (*PR*) gene expression as a transcriptional activator or repressor of GCC box-mediated gene expression and participates in plant defense regulation [14]. ERF6 acts as a substrate of MPK3/MPK6 and plays an important role in regulating the expression of defense genes as well as the defense against the necrotizing fungal pathogen *Botrytis cinerea* [15]. Knockdown of ethylene-responsive factor *CaPTI1* weakened the *Phytophthora capsici* defense by inhibiting the defense-related genes of *CaPR1*, *CaDEF1*, and *CaSAR82* in pepper [16]. GbERFb can bind with *cis*-acting elements of the GCC cassette and directly interact with GbMAPKb (MAP kinase) in yeast; overexpression of *GbERFb* increased the *V. dahliae* resistance in tobacco [17]. Likewise, overexpressing *GmERF113* positively regulates the expression of *PR1* and *PR10-1* genes, thereby enhancing the *Phytophthora sojae* resistance in soybean [18]. ZmERF105 is a transcriptional activator that binds with GCC box elements, and *ZmERF105* overexpression enhanced the disease resistance in maize [19]. The ERF.B4 group responds to drought, cold, and ABA, JA, ET, and SA signals [20]. ERF109, also known as redox-responsive TF 1 (RRTF1), is a key positive regulator in plant development and environmental adaptation. *ERF109* expression was induced with 4.3 (15 min) and 5.7 (30 min) fold responses to chitooctaose treatment [21]. RRTF1 interacts with the BTB/POZ-MATH (BPM) protein BPM1 and is involved in *Botrytis cinerea* resistance [22]. Transcriptome profiling analysis of *ERF109* mutations led to significant downregulation of defense genes, including *CML37*, *WRKY40*, *ERF13*, and *EXO70B2* [23].

The DREB (dehydration-responsive element binding) is a member of the ERF family. A total of 56 DREB members were detected in Arabidopsis, which could be divided into six subgroups: A1 (contains 6 members), A2 (contains 8 members), A3 (contains 1 member), A4 (contains 16 members), A5 (contains 16 members) and A6 (contains 9 members) [9]. The AP2 domain of the DREB transcription factor has high similarity between different species. However, various groups of DREB transcription factors have varying specificity for recognizing the downstream DRE/CRT motif core sequence A/GCCGAC [8]. Plant DREB transcription factors respond to various abiotic stresses. Additionally, DREB transcription factors are also sensitive to plant hormone signals and play an important role in transduction pathways involved in the signaling of various hormones [20]. DREB1B, another name for C-Repeat/DRE binding factor 1 (CBF1), is involved in the response to low temperatures and abscisic acid [24]. Expression of *OsDREB1B* was induced by glycerol, NaCl, PEG, and cold stresses [25]. Meanwhile, overexpression of *OsDREB1B* improved the abiotic and biotic stress resistances in tobacco [26]. CBF1 directly interacts with the GALACTAN SYNTHASE1 promoter to enhance salt tolerance by repressing the galactan accumulation [27]. A total of 183 *GhDREB* genes were identified in upland cotton; plenty of them were responsible for salt and osmotic stresses [28]. 

In the present study, we performed a comparative analysis of ERF.B4 group members in upland cotton. The evolutionary relationship, gene organization, conserved motif, and gene synteny analysis were developed. Meanwhile, the function of *GhERF.B4-15D* was examined by overexpression in Arabidopsis and interference expressed in cotton. Later, the protein interaction of GhERF.B4-15D was verified to understand the potential role of GhERF.B4-15D in *V. dahliae* resistance in upland cotton.

## 2. Materials and Methods

### 2.1. Identification and Phylogenetic Analysis of ERF Family B4 Group Members

The genome datasets of *G. arboretum* [29], *G. raimondii* [30], and *G. hirsutum* [31] were downloaded from the CottonGen website (https://www.cottongen.org/, accessed on 1 June 2023) [32]. The genome files of Arabidopsis were downloaded from The Arabidopsis Information Resource (TAIR) website (https://www.arabidopsis.org/, accessed on 20 May 2023). The blastp program was used to search with the amino acid sequences of AtERF108 (AT1G43160), AtERF109 (AT4G34410), AtERF110 (AT5G50080), AtERF111 (AT5G64750), AtERF112 (AT2G33710), AtERF113 (AT5G13330), AtERF114 (AT5G61890), and AtERF115 (AT5G07310) as queries to select ERF.B4 group members in cotton. Additionally, the AP2 domain (PF00847) was used to examine ERF members by using the Hidden Markov Model (HMM) through HMMER 3.0 software with a threshold value of 1e-5. The ERF.B4 genes were named based on the chromosomal location in cotton; “A” and “D” represent that these genes were located in the At and Dt subgenomes, respectively. The MUSCLE program in MEGA-11 software was used for multiple sequence alignment of ERF.B4 proteins, and a phylogenetic tree was constructed by the maximum likelihood (ML) method with 1000 bootstraps [33]. Subsequently, the phylogenetic tree was visualized by the iTOL website (https://itol.embl.de/, accessed on 8 July 2023) [34]. Further, the protein isoelectric point (pI) and theoretical molecular weight (MW) were evaluated by the ExPASy tool [35], and the subcellular localization of GhERF.B4 proteins was predicted by the Plant-mPLoc server [36].

### 2.2. Gene Structure, Conserved Domain and Motifs, and Gene Synteny Analysis

The organization of GhERF-B4 members on chromosomes was acquired from the genome database of upland cotton [31]. The conserved domains and motifs of GhERF-B4 proteins were detected through SMART and MEME tools, respectively [37,38]. Gene structure, the conserved domains, and motifs were displayed via the Gene Structure View program in TBtools software [39]. Additionally, Gene synteny analysis was performed by using the One Step MCScanX program of TBtools, and then the Advanced Circos program in TBtools software was used for graphical depiction [40].

### 2.3. Gene Expression Analysis

The RNA-Seq datasets of upland cotton were used to investigate the expression profiles of *GhERF.B4* genes [31]. Meanwhile, the expression data of upland cotton infected by *V. dahliae* was employed to view the disease response of GhERF.B4 members [41]. Gene expression levels were calculated following the previous description [5]. The VW-resistant cultivar Zhongzhimian 2 (Z2) was used for *GhERF.B4-15D* expression analysis in response to plant hormones. Cotton plants were treated with hormones described by Wu et al. (2022) [42]. The leaves were collected at six time point intervals, i.e., 0, 3, 6, 12, 24, and 48 h after treatments, and samples were instantly frozen in liquid nitrogen prior to total RNA extraction.

### 2.4. Overexpression Vector Construction and Transformed into A. thaliana

The coding sequence (CDS) of *GhERF.B4-15D* was amplified and connected to the pCAMBIA2300 vector (stored in our laboratory) to construct the recombinant vector p2300-GhERF.B4-15D driven by the CaMV 35S promoter (Table S1). The overexpression vector was transformed into *Arabidopsis thaliana* ecotype Columbia (Col-0) by the floral dip method [43]. The resistant plants were screened on 1/2 MS medium containing 50 mg/L kanamycin; total RNA was extracted and reverse transcribed into cDNA by using the PrimeScript™ RT reagent Kit (TaKaRa, Dalian, China). Then, reverse transcription-PCR (RT-PCR) was used to detect positive transgenic plants. The homozygous lines at T_3_ generation were further screened and used for *V. dahliae* infection.

### 2.5. Virus-Induced Gene Silencing (VIGS) in Upland Cotton

The upland cotton cultivar Z2 was used for the VIGS experiment. Short lint on the cotton seeds’ surface was removed with concentrated sulfuric acid. Then, the cotton seeds were sowed in nutritious soil and grown in the greenhouse (25 °C, light/darkness = 16 h/8 h, humidity = 80%). 

For functional characterization of GhERF.B4-15D and GhDREB1B, a specific 300 bp fragment of *GhERF.B4-15D* and *GhDREB1B* was amplified and inserted into the pTRV2 vector (stored in our laboratory) (Table S1). The recombinant vectors, TRV::GhERF.B4-15D and TRV::GhDREB1B, were transformed into *Agrobacterium tumefaciens* strain GV3101 (Weidi, Shanghai, China) by the freeze–thaw method following the instructions. When the cotyledons of cotton seedlings were fully established, *Agrobacterium* cultures containing TRV:GhERF.B4-15D and TRV:GhDREB1B were transformed into cotton leaves, respectively. Chlorophyll gene *CLA1* was used as a positive control, and the *Agrobacterium* mixture of pTRV1 and pTRV2 was used as a negative control. The *Agrobacterium* was resuspended to OD_600_ = 1.0 with the MMA resuspension solution (0.5 M MES, 1 M MgCl_2_, 200 mM AS). When true leaves of the positive plant (TRV::GhCLA) were photobleached, the root tissues of TRV::GhERF.B4-15D and TRV::GhDREB1B cotton plants were collected and taken for gene expression level analysis. The VIGS experiment was carried out three times with at least 48 plants per experiment.

### 2.6. V. dahliae Culture and Disease Resistance Analysis

*V. dahliae* was inoculated in PDB medium for 5 days and activated on a shaker at 25 °C, 220 rpm for 5 d. The number of Vd991 spores was counted under a microscope with a blood count plate, and the concentration of spores in solution was diluted to 10 × 10^6^ spores/mL with sterile water. The *A. thaliana* seeds were sowed in nutritious soil and grown in the greenhouse (22 °C, light/darkness = 16 h/8 h, humidity = 80%). In the experiment of inoculation, three-week-old seedlings of *A. thaliana* were pulled out of the soil and immersed in 1 × 10^6^ spores/mL liquid for 1 min. Then, the infected *A. thaliana* was replanted in the soil. The disease index was recorded after 21 days post inoculation (dpi).

For cotton inoculation, the concentration of spores in Vd991 solution was diluted to 1 × 10^7^ spores/mL with ddH_2_O. The roots of three-week-old cotton plants were carefully pulled out of the soil and completely immersed in Vd991 bacterial solution for 15 min. Then, infected seedlings were replanted in soil culture under normal conditions. The disease incidence was calculated by observing the degree of withered and yellow leaves. The disease index of cotton plants was calculated at 14, 21, and 28 dpi. The disease index (DI) was divided into 0–4 grades (0 = healthy plants or asymptomatic plants, 1 = diseased leaves accounted for 1–33%, 2 = 34–66%, 3 = 67–99%, 4 = dead plants). The calculation formula is as follows: DI = (∑[incidence level × number of plants at this level]/[Total number of plants investigated × 4]) × 100 [42].

### 2.7. Detection of Reactive Oxygen Species and Callose Deposition

The content of reactive oxygen species (ROS) in leaves was detected at 24 h after inoculation (hpi). The true leaves of cotton plants were washed with distilled water thoroughly, then placed in a 50 mL centrifuge tube and incubated with 3,3-diaminobezidine (DAB, 1 mg/mL, pH = 7.5) (Solarbio, Beijing, China) for 8 h at room temperature (25 °C). Thereafter, the DAB dye solution was removed, and 95% ethanol was added into the centrifuge tube and boiled for 2 min to elute the chlorophyll in the leaves. Then ethanol was removed from the tube, and 50 mL absolute ethanol was added in the same tubes until the color fading of the leaves was finished. Then, these decolorized leaves were shifted into tubes with 70% glycerol for observation under the microscope.

Meanwhile, cotton leaves were collected for callose deposition detection at 14 dpi. Cotton leaves were washed with distilled water and placed in a 50 mL centrifuge tube, and acetic acid–ethanol fixing solution (*v*:*v* = 1:3) was added to the tubes. The chlorophyll was removed after keeping at room temperature for 2–3 h; then, these samples were soaked in 70% and 50% ethanol for 2 h. Further, cotton leaves were treated with 10% NaOH for 1–2 h and stained with 0.01% aniline blue for 3–4 h. Then, the stained leaves were placed under a fluorescence microscope, and callose deposition was observed by UV light excitation (Leica, Weztlar, Germany).

### 2.8. V. dahliae Recovery Culture and Relative Fungal Biomass Detection

To view the browning degree of the cotton vascular bundle, TRV::00, TRV::GhERF.B4-15D, and TRV::GhDREB1B cotton plants were randomly selected, and stem segments were used for rod cutting at 14 dpi. Then, the browning degree of the vascular bundle in stem segments was observed under a microscope (Leica, Weztlar, Germany). Meanwhile, the fungus recovery assay was carried out on cotton plants at 14 dpi. Stem segments in the upper part of the cotyledon segment of TRV::00, TRV::GhERF.B4-15D, and TRV::GhDREB1B plants were cut off and sterilized by 20% NaClO for 10 min. Then, the stems were washed 3 to 5 times with sterilized ddH_2_O. Subsequently, the sterilized cotton stems were cut into 0.8 cm segments and placed in a PDA medium containing 50 mg/mL cephalosporin. The fungi growth in the stem segments was observed for preservation at 5 days after culture. To detect the fungal biomass, the fungal DNA of stem segments in TRV::00, TRV::GhERF.B4-15D, and TRV::GhDREB1B plants was extracted at 14 dpi. The fungal biomass of stem segments was detected using RT-qPCR by specific primers listed in Table S1.

### 2.9. Subcellular Localization

Specific primers were designed on restriction sites of K*pn* I and A*SC* I on the expression vector pCAMBIA2300-YFP (Table S1). Then, the amplified fragment of the *GhERF.B4-15D* gene was cloned and formed a fusion protein with YFP, which was named 35S::GhERF.B4-15D-YFP. The recombinant vector was transformed into *Agrobacterium tumefaciens* strain GV3101 and injected into tobacco (*Nicotiana benthamiana*) leaves. After 2–3 days, the tobacco leaves were cut off, and YFP fluorescence was observed on a laser confocal microscope with an ultraviolet spectrum excitation of 488 nm (Olympus, Tokyo, Japan).

### 2.10. Yeast Two-Hybrid and Luciferase Complementation Assays 

The full-length fragments of *GhERF.B4-15D* and *GhDREB1B* were cloned into pGBKT7 and pGADT7 vectors, respectively (Table S1). Firstly, the GhERF.B4-15D-BD vector was transformed into Y2HGold yeast competent cells (Coolaber, Beijing, China) following the manufacturer’s instructions to test the self-activation function on TDO medium (SD/-Trp/-Leu/-His). The yeast colonies will appear on the TDO plate if GhERF.B4-15D-BD exhibits transcriptional activation. The transcriptional self-activating activity was inhibited by 30 mM transcriptional activating inhibitors (3-amino-1,2,4-triazole, 3-AT) for the yeast two-hybrid (Y2H) experiment. GhERF.B4-15D-BD and GhDREB1B-AD vectors were co-transferred into yeast competent cells and cultured on a DDO medium (SD/-Trp/-Leu). Subsequently, the yeast colonies were diluted with sterile water and absorbed on QDO medium (SD/-Trp/-Leu/-His/-Ade + X-α-gal + 30 mM 3-AT) and incubated at 30 °C for 48~96 h.

To further verify the interaction relationship of GhERF.B4-15D and GhDREB1B, the CDS sequences of *GhERF.B4-15D* and *GhDREB1B* were cloned into nLUC and cLUC vectors to form GhERF.B4-15D-nLUC and GhDREB1B-cLUC, respectively (Table S1). Then, the different groups were transiently transformed into tobacco leaves for 48~72 h and photographed by an in vivo plant imaging system (NightSHADE LB 985, Berthold, Germany).

### 2.11. Real-Time Quantitative PCR and Data Statistical Analysis

Total RNA was extracted by using the RNAprep Pure Plant Kit (TIANGEN, Beijing, China), and first-strand DNA (cDNA) was synthesized using PrimeScript™ RT reagent Kit (TaKaRa, Dalian, China) referring to the manufacturer’s instructions. Then, RT-qPCR was performed using ChamQ Universal SYBR qPCR Master Mix (Vazyme, Nanjing, China) on a Light Cycler 480 machine (Roche, Basel, Switzerland). *GhUBQ7* and *AtActin* were used as internal references in upland cotton and Arabidopsis, respectively. Specific primers are listed in Table S1. The 2^−ΔΔCt^ method was used to calculate the relative expression levels of target genes. The experiment was performed in triplicate.

All statistical analyses were performed via Excel2020 and IBM SPSS Statistics 20.0 (SPSS, Chicago, IL, USA). The significant differences and asterisk marks were determined based on the Student’s *t*-test (*, *p* < 0.05, **, *p* < 0.01).

## 3. Results

### 3.1. Identification and Phylogenetic Analysis of ERF Subfamily B4 Group in Cotton

**Figure 1:**
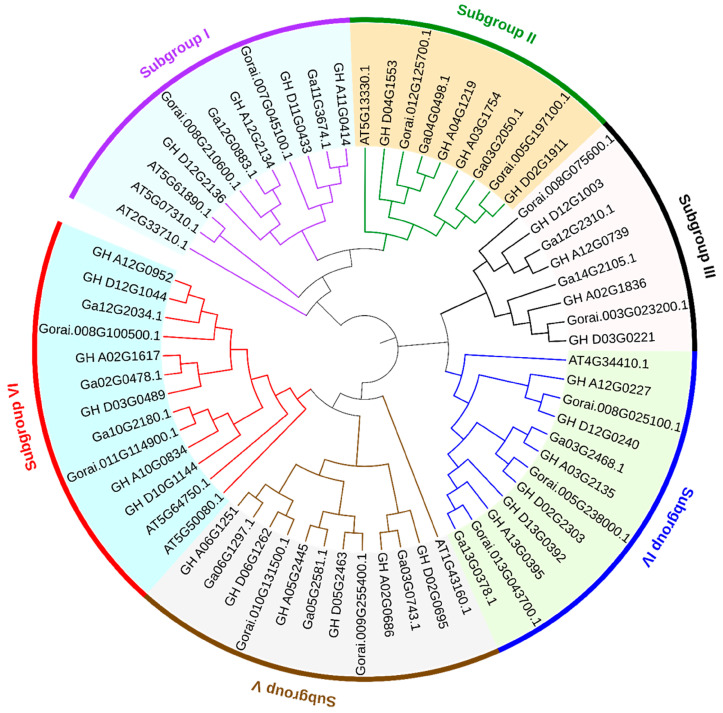
Phylogenetic analysis of the ERF.B4 group members in *A. thaliana*, *G. arboreum*, *G. raimondii*, and *G. hirsutum*.

A total of 14, 13, and 30 ERF subfamily B4 group members were detected in *G. arboreum*, *G. raimondii*, and *G. hirsutum*, respectively (Figure 1). An evolutionary tree was constructed according to the amino acid sequence of the ERF subfamily B4 group proteins. The results showed that they were divided into six subgroups. The number of ERF.B4 in tetraploid cotton (*G. hirsutum*) was about twice that compared to diploid cotton (*G. arboretum* and *G. raimondii*). At the same time, members in the At and A_2_ genomes (*G. arboreum*) tended to form one branch, while members in the Dt and D_5_ genomes (*G. raimondii*) tended to form one branch (Figure 1). These results confirmed that the tetraploid cotton species evolved through hybridization among diploid species of cotton, followed by genomic doubling. In addition, the phylogenetic tree showed that the ERF subfamily B4 group genes were conserved in cotton species and *A. thaliana.*

The 30 GhERF.B4 genes were distributed on 18 chromosomes. A12 and D12 chromosomes contain four GhERF.B4 members, respectively, which were more than the other chromosomes. Meanwhile, GhERF.B4 genes contain 1 to 3 exons, and the CDS length was from 546 bp (GhERF.B4-12D) to 1245 bp (GhERF.B4-7A) (Table 1). The physicochemical properties of GhERF.B4 proteins were also characterized. The pI ranged from 5.42 (GhERF.B4-12D) to 9.57 (GhERF.B4-9A and GhERF.B4-10A), while the MW ranged from 21.00 kDa (GhERF.B4-12D) to 44.72 kDa (GhERF.B4-7A) (Table 1). Subcellular localization showed that all the GhERF.B4 proteins were located in the nucleus, indicating that GhERF.B4 members are transcription factors (Table 1).

### 3.2. Gene Organization of GhERF.B4 Group

**Figure 2:**
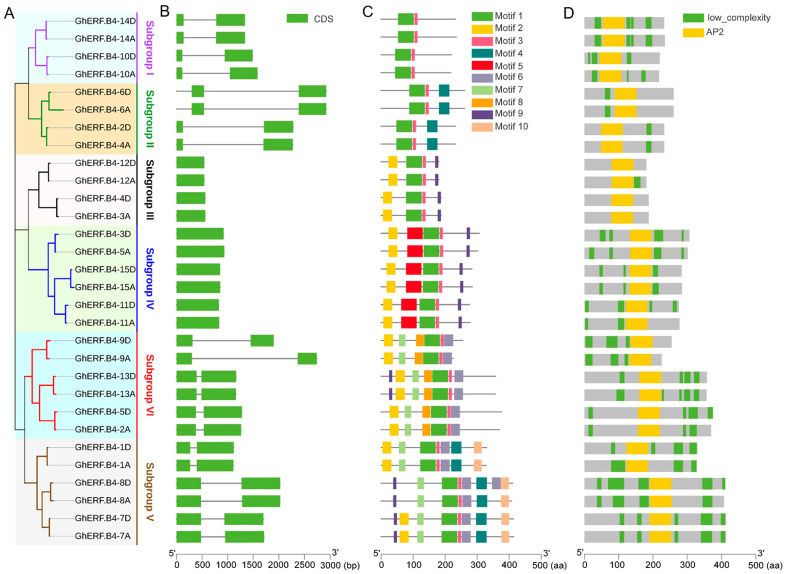
Gene structures and conserved motifs, domains of GhERF.B4 group members. (**A**) Phylogenetic tree of GhERF.B4 proteins; (**B**) The organization of exons and introns in *GhERF.B4* genes; (**C**) Conserved motifs of GhERF.B4 proteins; (**D**) Conserved domains of GhERF-B4 proteins.

An evolutionary tree was constructed to analyze the relationships among GhERF.B4 members. According to Figure 1, the 30 GhERF.B4 members were also divided into 6 subgroups (Figure 2A). The analysis of the gene structures exhibited that the homologous genes shared similar structures; members in subgroups III and IV contain one exon, while the members in subgroups I, II, V, and VI contain two to three exons (Figure 2B). Additionally, the 10 most conserved motifs were identified in the GhERF.B4 proteins. It is found that all GhERF.B4 proteins contain motif1 and motif3. In particular, subgroup II members contain motif4, subgroup III members contain motif2 and motif9, subgroup IV members contain motif2, motif5, and motif9, subgroup V members contain motif4, motif6, motif7, and motif10, and subgroup VI members contain motif2, motif6, motif7, and motif8. Overall, motifs in GhERF.B4 proteins of the same subgroups are highly conserved (Figure 2C). The motif annotation analysis showed that motif1 and motif3 belong to the AP2 domain, while other motifs do not belong to any conserved domains (Table S2). Protein-conserved domain prediction results revealed that all GhERF.B4 members have one AP2 domain (Figure 2D). In general, it was found that GhERF.B4 members in the same clade contain the same motifs, which was verified through evolutionary relationships.

### 3.3. Chromosomal Localization and Gene Synteny of GhERF.B4 Group

**Figure 3:**
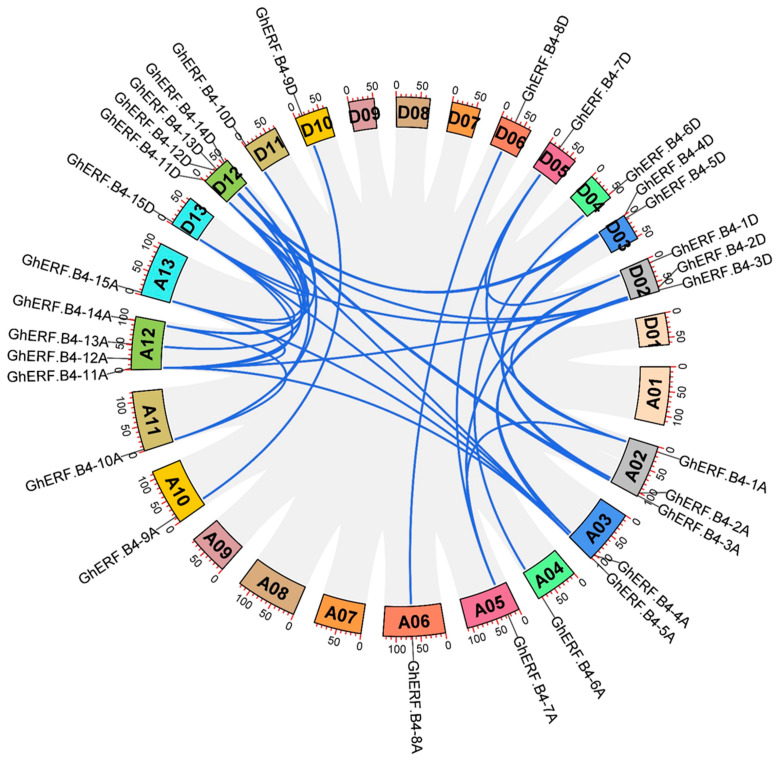
Syntenic relationships of GhERF.B4 members. Homologous chromosomes in the At and Dt subgenomes are displayed in the same color.

The 30 GhERF.B4 members were distributed on 18 chromosomes. There are four members, which are located on the A12 and D12 chromosomes, respectively, followed by three members located on the A02 and D02 chromosomes, respectively. Whereas two members are located on A03 and D03, and one *GhERF.B4* gene is distributed on A04, D04, A05, D05, A06, D06, A09, A10, D10, A11, D11, A13, and D13 chromosomes, respectively. Meanwhile, Gene synteny showed that 36 gene synteny relationships were found among 30 GhERF-B4 genes. Most GhERF-B4 genes were highly symmetric between At and Dt subgroups (Figure 3).

### 3.4. Expression Profiles of GhERF.B4

**Figure 4:**
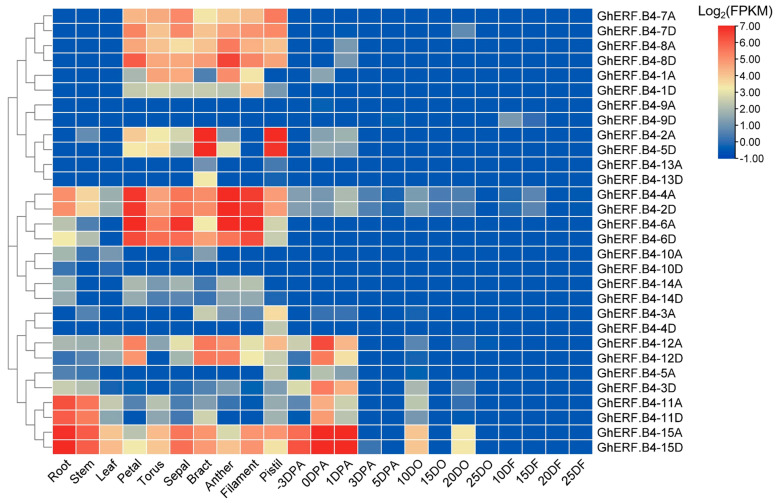
Expression profiles of *GhERF.B4* genes in upland cotton. DO, days post anthesis (DPA) ovules; DF, DPA fibers.

Gene expression profile is an important indicator of gene function. The transcript levels of *GhERF.B4* genes in various tissues and developmental stages of upland cotton were investigated using transcriptome datasets [31]. It was found that several *GhERF.B4* genes were abundant in petal, torus, sepal, bract, anther, filament, and pistil tissues. Meanwhile, we observed that most of them were barely expressed in developing ovules and fiber. However, the homologous genes of *GhERF.B4-2A/5D* in subgroup II were highly expressed in bract and pistil, while the duplicated genes *GhERF.B4-13A/D* were elevated in bract, whereas the duplicated genes *GhERF.B4-9A/D* were not expressed in cotton tissues (Figure 4). Simultaneously, we noticed that *GhERF.B4-15A/D* was highly expressed in the roots, stems, sepals, and early ovules (−3, 0, and 1 DPA), while it was barely expressed in fiber (Figure 4).

**Figure 5:**
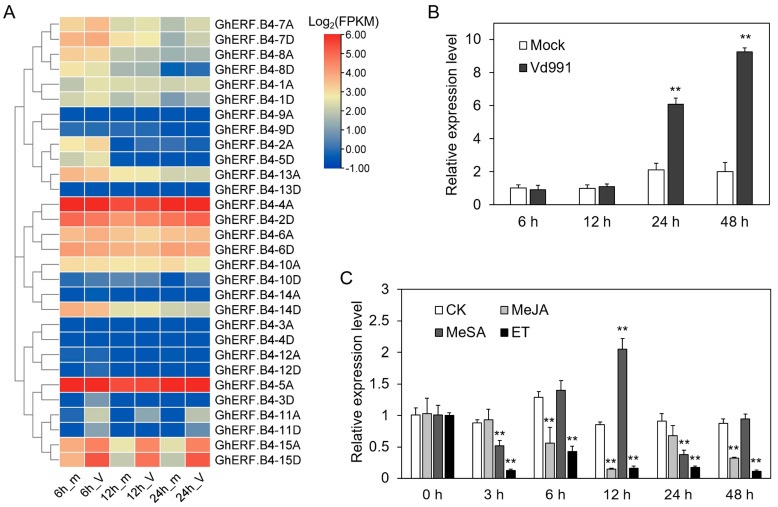
Expression analysis of *GhERF.B4-15D* under *V. dahliae* infection and plant hormones treatments. (**A**) Expression patterns of *GhERF.B4* genes in response to *V. dahliae* by RNA-seq data. m, mock; V, Vd991. (**B**) The transcript level of *GhERF.B4-15D* under *V. dahliae* infection was tested by RT-qPCR. (**C**) *GhERF.B4-15D* response to MeSA, MeJA, and ET hormones. “**” indicate *p* < 0.01.

To evaluate the potential role of *GhERF.B4* in response to biotic stress, expression levels of *GhERF.B4* genes were calculated by transcriptome datasets of upland cotton infected by *V. dahliae* strain Vd991. It was found that homologous genes *GhERF.B4-15A/D* were induced upon *V. dahliae* infection (Figure 5A). Meanwhile, RT-qPCR analysis showed that the expression of *GhERF.B4-15A/D* was significantly upregulated after 24 h and 48 h of inoculation with *V. dahliae* (Figure 5B). To assess the response of *GhERF.B4-15A/D* to plant hormones, the expression patterns of *GhERF.B4-15A/D* were determined under MeJA, MeSA, and ET treatments. *GhERF.B4-15A/D* exhibited downregulation at 6, 12, and 48 h under MeJA treatment, at 3 and 24 h under MeSA treatment, and at all time points under ET treatment. However, the expression of *GhERF.B4-15A/D* was upregulated at 12 h of MeSA treatment (Figure 5C).

### 3.5. Overexpression of GhERF.B4-15D Confers V. dahliae Resistance in A. thaliana

**Figure 6:**
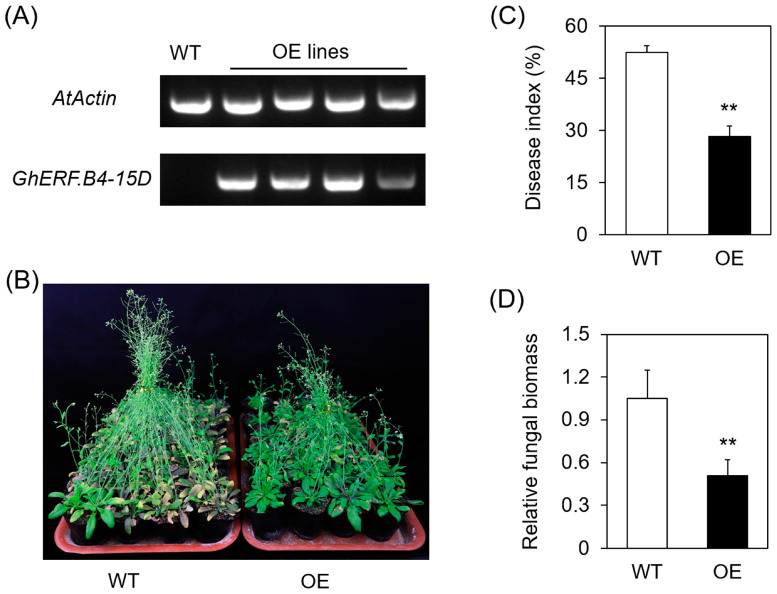
Overexpression of *GhERF.B4-15D* increased *V. dahliae* resistance in *A. thaliana*. (**A**) Detection of the positive plants by RT-PCR. (**B**) The phenotype of wild-type (WT) and transgenic (OE) plants after *V. dahliae* infection. (**C**,**D**). Statistical analysis of the disease index and fungal biomass in rosette leaves. **, *p* < 0.01. All experiments were repeated three times.

*GhERF.B4-15D* expression was more induced than that of *GhERF.B4-15A* upon *V. dahliae* infection (Figure 5A). Thus, GhERF.B4-15D was selected for functional analysis. The 35S::GhERF.B4-15D vector was transformed into *A. thaliana* using the floral dip method. RT-PCR results showed that *GhERF.B4-15D* was successfully expressed in transgenic Arabidopsis (Figure 6A). The single-copy insertion homozygous transgenic lines in the T_3_ generation were used for *V. dahliae* infection. It was found that the *GhERF.B4-15D* overexpressing (OE) lines exhibited more green leaves and relatively fewer yellowed leaves compared with the wild-type at 21 dpi, implying that the *V. dahliae* resistance was enhanced in transgenic lines (Figure 6B). Meanwhile, the disease index and fungal biomass in the transgenic line were significantly lower than in the wild-type (Figure 6C,D). Therefore, these results suggest that overexpression of *GhERF.B4-15D* increases the *V. dahliae* resistance in Arabidopsis.

### 3.6. Interference of GhERF.B4-15D Decreases the V. dahliae Resistance in Upland Cotton

**Figure 7:**
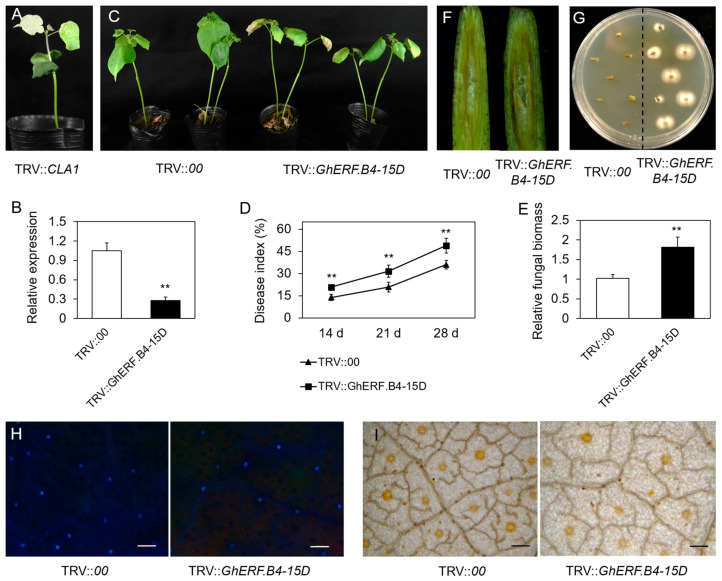
Knockdown of *GhERF.B4-15D* decreased *V. dahliae* resistance in upland cotton. (**A**) The photobleached phenotype in the TRV::CLA1 line. (**B**) Expression levels of *GhERF.B4-15D* in TRV::00 and TRV::GhERF.B4-15D cotton plants. (**C**) Phenotypes of TRV::00 and TRV::GhERF.B4-15D plants. (**D**,**E**). Statistics of disease index and relative fungal biomasses in control and *GhERF.B4-15D* interfering lines. (**F**) The browning degree in stem vascular bundles. (**G**) *V. dahliae* recovery culture assay. (**H**,**I**) Callose deposition and ROS accumulation in TRV::00 and TRV::GhERF.B4-15D cotton leaves. Bar = 200 μm. **, *p* < 0.01.

To determine the role of *GhERF.B4-15D* in resistance to *V. dahliae* in cotton, the expression of *GhERF.B4-15D* was interfered with by the VIGS method. The photobleaching phenotype appeared in the positive control at 10 days post-infection (TRV::CLA1) (Figure 7A). The RT-qPCR approach detected that the expression of *GhERF.B4-15D* was knocked down in TRV::GhERF.B4-15D lines (Figure 7B). Then, *GhERF.B4-15D* silencing and control plants were inoculated with Vd991. We observed more yellowing and wilting symptoms in TRV::GhERF.B4-15D plants, which were more sensitive to *V. dahliae* as compared to control plants (Figure 7C). The disease index in TRV::GhERF.B4-15D plants was higher than TRV::00 plants at 14, 21, and 28 dpi (Figure 7D). In addition, fungal DNA was extracted from TRV::GhERF.B4-15D and TRV:00 cotton plants; the results showed that the fungal biomass in TRV::GhERF.B4-15D plants was increased as compared to control (Figure 7E). Furthermore, it was found that vascular bundles exhibited more browning and blockage in GhERF.B4-15D-silenced cotton plants than in the control (Figure 7F). Studies on fungal recovery showed that higher fungal colonization appeared in TRV::GhERF.B4-15D plants than in TRV::00 plants (Figure 7G). 

The production of ROS and deposition of callose play an important role in defense responses against pathogen invasion [44]. Therefore, DAB staining was performed on TRV::00 and TRV::GhERF.B4-15D cotton leaves to detect ROS levels. It was found that GhERF.B4-15D-silenced plants had significantly less accumulation of ROS as compared to control plants (Figure 7H). In addition, GhERF.B4-15D-silenced lines exhibited less deposition of callose after infection with Vd991 as compared to TRV::00 plants (Figure 7I). These results suggest that interference of *GhERF.B4-15D* reduces the *V. dahliae* resistance in upland cotton.

### 3.7. GhERF.B4-15D Localized in Nucleus and Interacted with GhDREB1B

**Figure 8:**
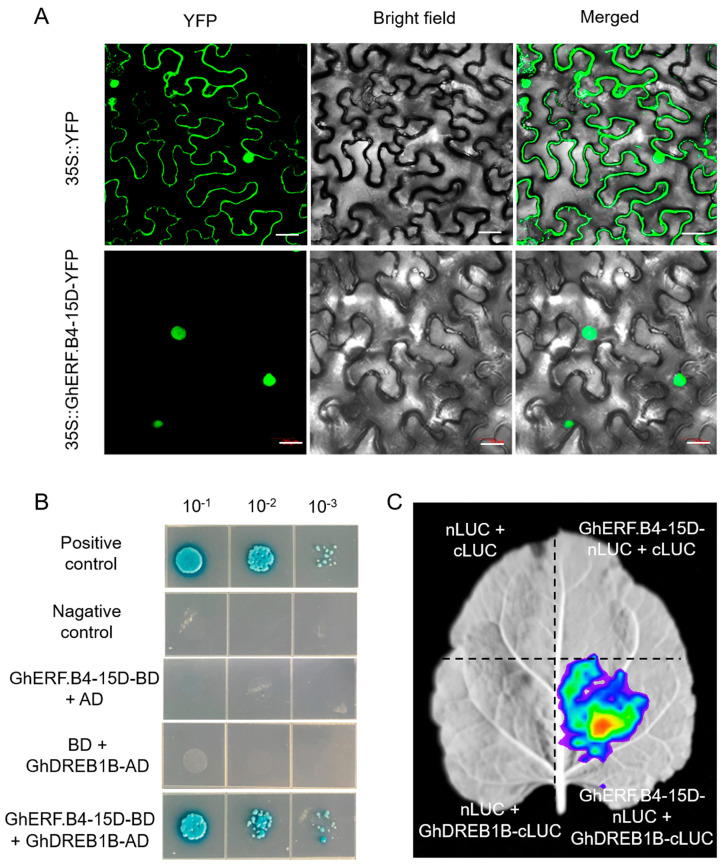
Subcellular localization of GhERF.B4-15D and the interaction of GhERF.B4-15D and GhDREB1B. (**A**) Subcellular localization of GhERF.B4-15D. YFP, Yellow fluorescent protein, bar = 20 μm. (**B**) GhERF.B4-15D interacted with GhDREB1B by yeast two-hybrid assay. The transformants were grown on SD/-Leu/-Trp/-Ade/-His (+ X-α-gal and 30 mM 3-AT) media. pGBKT7-53/pGADT7-T was used as the positive control, pGBKT7-Lam/ pGADT7-T were used as negative control. (**C**) Detect the interaction of GhERF.B4-15D with GhDREB1B by LUC. The *N. benthamiana* leaves were exposed using an in vivo plant imaging system (NightSHADE LB 985, Berthold, Germany).

The fusion vector of 35S::GhERF.B4-15D-YFP was constructed and transiently transformed to tobacco leaves to determine the subcellular localization of GhERF.B4-15D. The fluorescent protein was observed through a laser scanning confocal microscope at 2 d after inoculation, and green fluorescence was observed in the cell membrane, cytoplasm, and nucleus of 35S::YFP tobacco leaves, while green fluorescence was observed only in the nucleus of 35S::GhERF.B4-15D-YFP tobacco leaves (Figure 8A). The assay indicates that GhERF.B4-15D was located in the nucleus, according to the prediction of subcellular localization (Table 1).

A bait vector GhERF.B4-15D-BD was constructed to verify the interaction protein of GhERF.B4-15D by the yeast two-hybrid approach. Firstly, the GhERF.B4-15D-BD vector was subjected to self-activation, while the yeast colony was observed in the SD/-Trp/-Ade/-His medium (Figure S1). The assay showed that GhERF.B4-15D-BD was self-activated, and it might have transcriptional activity. GhERF.B4-15D exhibited the typical characteristics of a transcription factor upon incorporation with the nucleus localization. The self-activation of GhERF.B4-15D-BD was inhibited by 30 mM self-activation inhibitor 3-AT (3-amino-1, 2, 4-triazole). The co-expression network analysis showed that *GhERF.B4-15D-BD* was co-expressed with *GhDREB1B* in upland cotton. In addition, *GhDREB1B* expression was also induced by *V. dahliae* infection (Figure S2). Thus, the interaction between GhERF.B4-15D and GhDREB1B was verified by the Y2H approach. The results showed that yeast cells co-transfected with GhERF.B4-15D-BD and GhDREB1B-AD were observed on a deficient medium (SD/-Trp/-Leu-Ade/-His + X-α-gal), indicating that GhERF.B4-15D interacts with GhDREB1B (Figure 8B). Additionally, the interaction between GhERF.B4-15D and GhDREB1B was further verified by luciferase complementation assays. *GhERF.B4-15D* and *GhDREB1B* were connected to nLUC and cLUC luciferase vectors, respectively, and various combination lines were transiently transformed into tobacco leaves. This assay showed that the fluorescent signals only existed in the GhERF.B4-15D-BD-nLUC/GhDREB1B-cLUC line, while fluorescence was not seen in the negative controls (Figure 8C). This result confirmed that GhERF.B4-15D interacts with GhDREB1B.

### 3.8. V. dahliae Resistance Level Was Weakened through Silencing of GhDREB1B

**Figure 9:**
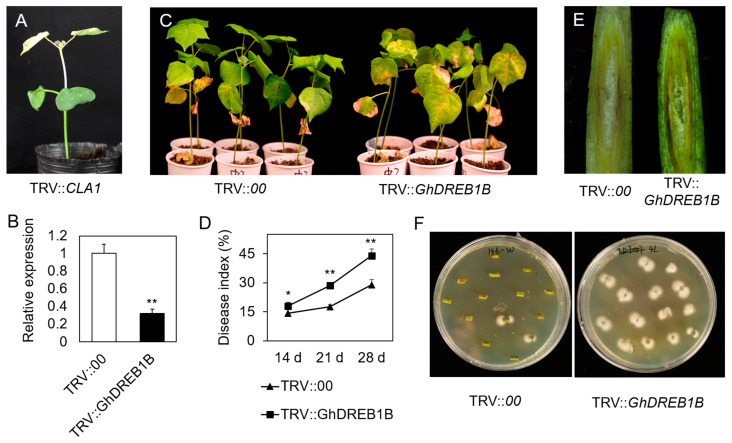
Downregulation of *GhDREB1B* weakened *V. dahliae* resistance in upland cotton. (**A**) The TRV::CLA1 photobleached phenotype. (**B**) *GhDREB1B* interfered expression in TRV::GhDREB1B plants. (**C**) The *Verticillium* wilt phenotypes in TRV::00 and TRV::GhDREB1B cotton. (**D**) Calculation of the disease index at 14, 21, and 28 dpi after *V. dahliae* infection. (**E**) Investigation of the browning degree in stem vascular bundles. (**F**) The degree of fungal colonization in TRV::00 and TRV::GhDREB1B plants. *, *p* < 0.05; **, *p* < 0.01.

To verify the role of GhDREB1B in cotton resistance to *V. dahliae*, a VIGS vector of GhDREB1B (TRV::GhDREB1B) was constructed for infection in cotton. When TRV::CLA plants showed a photobleaching phenotype (Figure 9A), the RT-qPCR approach was performed, and it was shown that *GhDREB1B* was effectively knocked down in the TRV::GhDREB1B lines (Figure 9B). Then, the different cotton lines were infected with Vd991. We observed that TRV::GhDREB1B plants were more susceptible to *V. dahliae* than control; symptoms included more wilting and yellowing of leaves (Figure 9C). The disease index was 18.06, 28.41, and 44.16 in *GhDREB1B*-silenced plants at 14, 21, and 28 dpi, which were significantly higher than in control plants (the disease index was 14.22, 17.64, and 29.22 at 14, 21, and 28 dpi, respectively) (Figure 9D). In addition, the degree of vascular browning of *GhDREB1B*-silenced plants was significantly lower than control plants at 21 dpi (Figure 9E). The fungal colonization in *GhDREB1B*-silenced plants was higher than the control (Figure 9F). Those results demonstrated that silencing of *GhDREB1B* increased the susceptibility of cotton against *V. dahliae*.

## 4. Discussion

### 4.1. Structure and Evolutionary Analysis of ERF.B4 Group

A total of 220 GhERF subfamily genes were detected in *G. hirsutum*, and some of them were selected as candidate genes against salinity tolerance [45]. Meanwhile, the ERF B3 group gene family was described, and *GhERF13.12* was expected to have a role in salt stress tolerance in Arabidopsis and upland cotton [46]. However, the B4 group in the GhERF subfamily has not been fully dissected and analyzed. In this study, the evolutionary relationship and expression pattern of GhERF.B4 in cotton were analyzed. The heterotetraploid species (*G. hirsutum*) were produced by crossing two diploid cottons (*G. arboreum* and *G. raimondii*) [47]. In total, 57 *ERF-B4* genes were identified in three species of cotton, and approximately twice the number of *ERF.B4* genes were found in *G. hirsutum* (30 genes) as in *G. arboreum* (14 genes) and *G. raimondii* (13 genes) (Figure 1). This proved that heterotetraploid cotton species evolved through hybridization from two diploid species.

According to phylogenetic analysis, ERF.B4 proteins can be divided into six subgroups. The clustering of genes from the same subgroup showed a high degree of sequence homology. GhERF.B4 has a similar exon and intron structure in one GhERF.B4 clade. In addition, the same subgroup of GhERF.B4 proteins had conserved protein motifs, which was consistent with the results of the gene structure analysis (Figure 2). The collinearity analysis result showed that GhERF.B4 genes in *G. hirsutum* had strong collinearity (Figure 3). According to the location of the GhERF.B4 members on the chromosomes, segmental duplication may have facilitated the evolution and the expansion of GhERF.B4 members.

### 4.2. Role of GhERF.B4 in Plant Development

ERF transcription factors play an important role in plant growth and development [7]. It was found that ERF115, ERF114, and ERF109 mediate ROS signaling and control the maintenance of root stem cell niche and root growth of Arabidopsis through plant sulfur factor (PSK) polypeptide hormones [48]. In this study, we reported that GhERF.B4 genes were broadly expressed in various tissues of upland cotton. Meanwhile, it was present that two copies of genes belonging to the same subgroup were expressed consistently in various tissues, providing the clue of a similar role in developmental phases in cotton (Figure 4). However, some duplicated genes exhibited diverse expression profiles, indicating that multi-functionalization, sub-functionalization, and non-functionalization events occurred during the evolution of the GhERF.B4 group (Figure 4). Among them, *GhERF.B4-15D* was abundantly expressed in early-developing ovules (−3, 0, and 1 DPA) but was barely expressed in fibers, implying that GhERF.B4-15D may play a role in embryogenesis (Figure 4). ERF109 was involved in lateral root formation by mediating the crosstalk between JA and the auxin pathway in Arabidopsis [49]. Meanwhile, ERF109 induces root stem cell activation and activates growth under environmental stress [50]. We found that GhERF.B4-15D was highly expressed in cotton roots, which indicated that GhERF.B4-15D had a role in root development and microbial interaction (Figure 4). 

### 4.3. GhERF.B4-15D Participate in Disease Resistance

The extensive role of ERF transcription factors in responding to multiple abiotic stresses has been fully discussed in plants [10]. However, ERF is also involved in the interaction of plants and pathogens [51,52]. Hormones play an important role in resistance to various pathogens. In this study, we have analyzed the expression profiles of *GhERF.B4-15D* under MeJA, MeSA, and ET hormones, where *GhERF.B4-15D* exhibited decreased expression under MeJA and ET treatments and showed fluctuant expression profiles under MeSA treatment, indicating that *GhERF.B4-15D* might be involved in hormone-mediated immunity responses (Figure 5C). However, the fluctuant expression patterns of *GhERF.B4-15D* under MeSA treatment might be due to the complex process of immune response in plants.

ERF transcription factors trigger defense responses and protect plants from pathogens by inhibiting or activating related gene expression, osmolyte, or chitinase transcription [51]. Previous studies have shown that ERF1 and its homologs belong to the ERF-IX class, and they were widely characterized in pathogen response involvement in Arabidopsis [53]. Tobacco OPBP1 increases the resistance to pathogenic bacteria when ectopically expressed in transgenic rice [54]. Arabidopsis RAP2.2 plays a role in plant ethylene resistance and response to *Botrytis cinerea* [55]. OsERF922 negatively regulates the resistance to *M. oryzae* in rice [56]. In recent years, it was reported that RRTF1 interacted with BTB/POZ-MATH (BPM) protein and response to *Botrytis cinerea* [22]. Knockout of *ERF109* downregulated the expression of defense genes of *CML37*, *WRKY40*, *ERF13*, and *EXO70B2* [23]. In this study, we found that the expression level of *GhERF.B4-15D* was induced at 24 and 48 h after inoculation with *V. dahliae*. Transgenic *A. thaliana* showed mild symptoms, including yellow leaves, withered leaves, and wilting after infection with *V. dahliae* (Figure 6). The expression of *GhERF.B4-15D* was interfered with in upland cotton, and it was found that the TRV::GhERF.B4-15D plants exhibited more yellowing and defoliation leaves. The ROS accumulation and callose deposition were reduced, and fungal biomass was more colonized in TRV::GhERF.B4-15D cotton leaves than in control (Figure 7). In conclusion, GhERF.B4-15D positively regulates the resistance against *V. dahliae* in Arabidopsis and upland cotton.

### 4.4. Interaction between GhERF.B4-15D and GhDREB1B

GmDREB1 was revealed as drought-inducible by interacting with two ERF transcription factors, GmERF008 and GmERF106 [57]. In this study, it was verified that GhERF.B4-15D interacts with GhDREB1B in the Y2H assay, and interaction between them was further verified by the LUC approach (Figure 8). So, both GhERF.B4-15D and GhDREB1B positively regulate the *V. dahliae* resistance and participate in the defense response of cotton against *V. dahliae* (Figure 7 and Figure 9). However, the interacting mechanism of GhERF.B4-15D and GhDREB1B and how they recognize pathogens to regulate cotton resistance to *V. dahliae* need further research.

## 5. Conclusions

The B4 group member in the ERF subfamily was comprehensively analyzed here, and *GhERF.B4-15D* expression was elevated when infected by *V. dahliae*. The *V. dahliae* resistance was enhanced by overexpressing of *GhERF.B4-15D* in *A. thaliana* and compromised by *GhERF.B4-15D* interfering in upland cotton. GhERF.B4-15D protein was localized in the nucleus and interacted with GhDREB1B. Our study revealed that GhERF.B4-15D positively regulates *V. dahliae* resistance, and it can be used to produce *V. dahliae*-resistant varieties for breeders in upland cotton.

## Figures and Tables

**Table 1:** Information of GhERF.B4 members in *G. hirsutum*.

Gene Name	Gene ID	Position	Exons	CDS (bp)	Amino Acid (aa)	pI	MW (kDa)	Subcellular Localization
*GhERF.B4-1A*	*GH_A02G0686*	A02: 10183281-10184396	2	990	329	6.49	36.00	Nucleus
*GhERF.B4-2A*	*GH_A02G1617*	A02: 100771344-100772609	2	1113	370	5.54	40.08	Nucleus
*GhERF.B4-3A*	*GH_A02G1836*	A02: 106309070-106309636	1	567	188	6.02	21.70	Nucleus
*GhERF.B4-4A*	*GH_A03G1754*	A03: 103081139-103083415	2	702	233	5.73	26.15	Nucleus
*GhERF.B4-5A*	*GH_A03G2135*	A03: 109300815-109301750	1	909	302	8.33	32.76	Nucleus
*GhERF.B4-6A*	*GH_A04G1219*	A04: 80131548-80134477	3	786	261	5.52	50.09	Nucleus
*GhERF.B4-7A*	*GH_A05G2445*	A05: 24877493-24879208	2	1245	414	6.42	44.72	Nucleus
*GhERF.B4-8A*	*GH_A06G1251*	A06: 67305332-67307360	2	1227	408	6.08	43.77	Nucleus
*GhERF.B4-9A*	*GH_A10G0834*	A10: 13232915-13235660	2	681	226	9.57	25.13	Nucleus
*GhERF.B4-10A*	*GH_A11G0414*	A11: 3664746-3666331	2	657	218	9.57	24.24	Nucleus
*GhERF.B4-11A*	*GH_A12G0227*	A12: 3229214-3230050	1	837	278	6.01	30.35	Nucleus
*GhERF.B4-12A*	*GH_A12G0739*	A12: 22078594-22079139	1	546	181	5.99	21.01	Nucleus
*GhERF.B4-13A*	*GH_A12G0952*	A12: 51497574-51498739	2	1074	357	5.94	39.57	Nucleus
*GhERF.B4-14A*	*GH_A12G2134*	A12: 99390602-99391942	2	708	235	9.1	26.02	Nucleus
*GhERF.B4-15A*	*GH_A13G0395*	A13: 4812697-4813554	1	858	285	5.87	31.63	Nucleus
*GhERF.B4-1D*	*GH_D02G0695*	D02: 9423518-9424640	2	993	330	6.63	36.23	Nucleus
*GhERF.B4-2D*	*GH_D02G1911*	D02: 62645157-62647441	2	702	233	6.07	26.13	Nucleus
*GhERF.B4-3D*	*GH_D02G2303*	D02: 67537107-67538030	1	924	307	7.62	33.41	Nucleus
*GhERF.B4-4D*	*GH_D03G0221*	D03: 1888772-1889338	1	567	188	5.59	21.43	Nucleus
*GhERF.B4-5D*	*GH_D03G0489*	D03: 6766278-6767559	2	1131	376	5.74	40.92	Nucleus
*GhERF.B4-6D*	*GH_D04G1553*	D04: 50223152-50226081	3	786	261	6.31	29.30	Nucleus
*GhERF.B4-7D*	*GH_D05G2463*	D05: 22462110-22463811	2	1245	414	6.42	44.70	Nucleus
*GhERF.B4-8D*	*GH_D06G1262*	D06: 29825029-29827056	2	1239	412	6.32	44.26	Nucleus
*GhERF.B4-9D*	*GH_D10G1144*	D10: 15035798-15037699	2	768	255	9.34	27.90	Nucleus
*GhERF.B4-10D*	*GH_D11G0433*	D11: 3394571-3396061	2	663	220	9.16	24.26	Nucleus
*GhERF.B4-11D*	*GH_D12G0240*	D12: 3025298-3026128	1	831	276	6.14	30.10	Nucleus
*GhERF.B4-12D*	*GH_D12G1003*	D12: 30662721-30663266	1	546	181	5.42	21.00	Nucleus
*GhERF.B4-13D*	*GH_D12G1044*	D12: 33281303-33282471	2	1077	358	5.55	39.47	Nucleus
*GhERF.B4-14D*	*GH_D12G2136*	D12: 53715169-53716508	2	702	233	8.78	25.79	Nucleus
*GhERF.B4-15D*	*GH_D13G0392*	D13: 4168311-4169165	1	855	284	5.99	31.48	Nucleus

## Data Availability

Not applicable.

## Supplementary Materials

The following supporting information can be downloaded at: https://www.mdpi.com/article/10.3390/biom13091348/s1. Table S1: Primers used in the present study. Table S2: The amino acid sequences and the annotation of the conserved motifs in GhERF.B4 proteins. Figure S1: Expression patterns of *GhDREB1B* by *V. dahliae* infection. Figure S2: Detection of the transcriptional activation effect of GhERF.B4-15D-BD in yeast.
