# Supplementary figures and images for: GhERF.B4-15D: A Member of ERF Subfamily B4 Group Positively Regulates the Resistance against Verticillium dahliae in Upland Cotton
Source: Biomolecules. 2023 Sep 5;13(9):1348. doi: 10.3390/biom13091348 (PMC10526341; doi:10.3390/biom13091348)

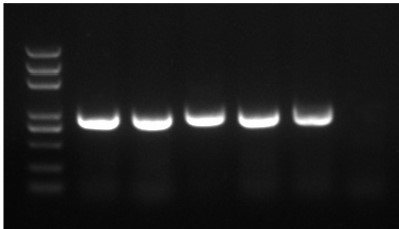

Supplement: Supplementary file 1 [file biomolecules-13-01348-s001.zip › biomolecules-2580063-Fig. 6A-AtActin.jpg]

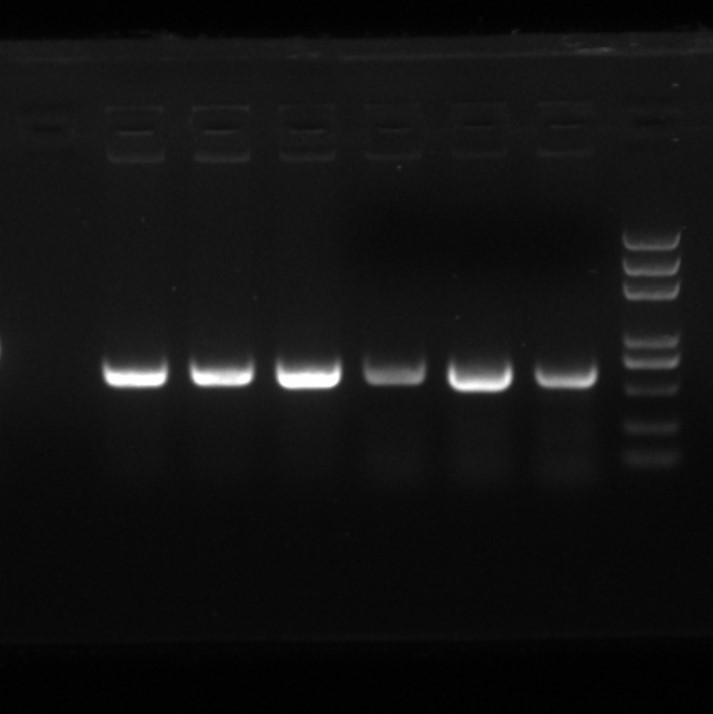

Supplement: Supplementary file 1 [file biomolecules-13-01348-s001.zip › biomolecules-2580063-Fig. 6A-GhERF.B4-15D-OE.jpg]

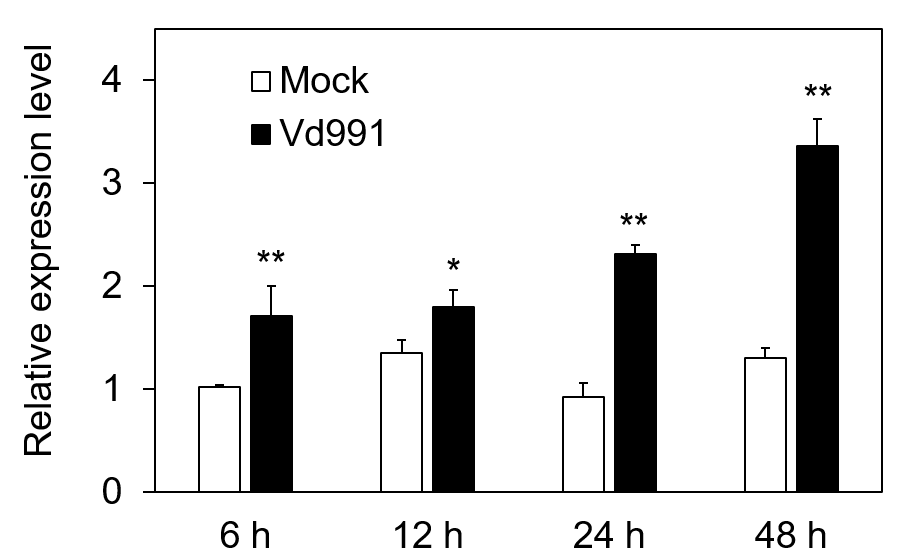

Supplement: Supplementary file 1 [file biomolecules-13-01348-s001.zip › Figure S1.tif]

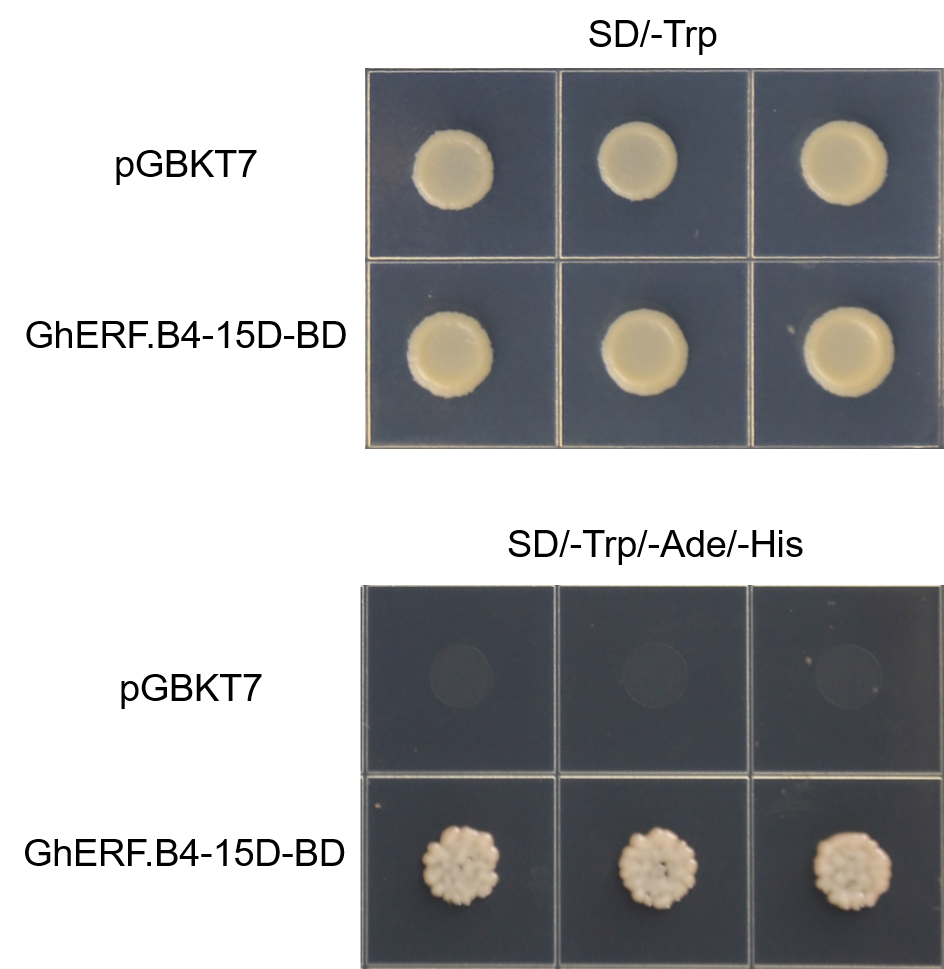

Supplement: Supplementary file 1 [file biomolecules-13-01348-s001.zip › Figure S2.tif]
